# Video Capsule Endoscopy Plays an Important Role in the Management of Crohn’s Disease

**DOI:** 10.3390/diagnostics13081507

**Published:** 2023-04-21

**Authors:** Asaf Levartovsky, Rami Eliakim

**Affiliations:** Department of Gastroenterology, Sheba Medical Center, Sackler School of Medicine, Tel Aviv University, Tel-Aviv 69978, Israel

**Keywords:** treat-to-target, PillCam Crohn’s capsule (PCC), pan-enteric, mucosal healing, post-operative relapse

## Abstract

Crohn’s disease (CD) is a chronic inflammatory disorder characterized by a transmural inflammation that may involve any part of the gastrointestinal tract. An evaluation of small bowel involvement, allowing recognition of disease extent and severity, is important for disease management. Current guidelines recommend the use of capsule endoscopy (CE) as a first-line diagnosis method for suspected small bowel CD. CE has an essential role in monitoring disease activity in established CD patients, as it can assess response to treatment and identify high-risk patients for disease exacerbation and post-operative relapse. Moreover, several studies have shown that CE is the best tool to assess mucosal healing as part of the treat-to-target strategy in CD patients. The PillCam Crohn’s capsule is a novel pan-enteric capsule which enables visualization of the whole gastrointestinal tract. It is useful to monitor pan-enteric disease activity, mucosal healing and accordingly allows for the prediction of relapse and response using a single procedure. In addition, the integration of artificial intelligence algorithms has showed improved accuracy rates for automatic ulcer detection and the ability to shorten reading times. In this review, we summarize the main indications and virtue for using CE for the evaluation of CD, as well as its implementation in clinical practice.

## 1. Introduction

Capsule endoscopy (CE) was first introduced and used in 2000 for the evaluation of the small intestine [1]. Before its introduction, the small bowel was difficult to assess using endoscopy. While upper endoscopy enabled direct visualization of the duodenum and lower endoscopy reached the terminal ileum, most of the small bowel remained outside of the reach of conventional endoscopy.

Enteroscopy allows for the endoluminal examination of the jejunum, yet usually not the full extent of the small bowel [2]. Different enteroscopic techniques are currently used for the evaluation of the small bowel. These techniques are known as device-assisted enteroscopy. The double-balloon enteroscopy, certainly the most established deep enteroscopy technique, was introduced in 2001 [3]. Additional methods were later introduced, such as single-balloon enteroscopy, spiral enteroscopy and a motorized spiral enteroscopy [4,5]. Imaging modalities, such as small bowel follow-through (SBFT), CT enterography (CTE) and MR enterography (MRE), are additional tools for the evaluation of the small bowel. However, these modalities may not detect mild and subtle lesions, which can be easily and accurately visualized with CE [6,7]. Therefore, CE has revolutionized the assessment of small-bowel mucosa as it is a non-invasive accurate diagnostic modality [8,9]. CE is currently the first-line of choice for patients with obscure gastrointestinal bleeding when no source has been identified by upper or lower endoscopy for the evaluation of small-bowel tumors and the surveillance of inherited polyposis syndromes [10]. Moreover, it has been increasingly used for the evaluation and management of inflammatory bowel disease (IBD), mainly Crohn’s disease (CD) [11]. CD is a chronic inflammatory disorder that may involve the entire gastrointestinal tract, but most commonly affects the small bowel. Disease location varies among patients, with small intestine involvement in 70–90% of patients, with up to 30% of patients having exclusive small bowel disease [12,13,14]. CD is characterized by a transmural inflammation potentially leading to various complications, including abscess, fistula, perforation and stricture. Thus, the evaluation of a small bowel disease, allowing early diagnosis, is necessary for the management of CD. Generally, CE, MRE, CTE and intestinal ultrasound are considered the preferred methods for small bowel evaluation in CD. CE is useful in assessing disease activity and extent, monitoring the treat-to-target effect of therapy and post-operative recurrence in patients with established CD. Current guidelines recommend the use of CE for the assessment of disease location and activity in patients with suspected or established CD [15,16]. In addition, it is used for the evaluation of response to therapy, the assessment of mucosal healing and suspected relapse [17,18]. Artificial intelligence (AI) is a promising integral technology in many areas of medicine, including gastroenterology and IBD [19]. An increasing number of studies have been published regarding the potential of AI in the field of CE. This implementation has the potential to assist in the detection of lesions and facilitate reading times, becoming a new standard for automated detection in CD. We conducted a literature review and searched PubMed for all publications on the subject of CE in IBD up to the year 2022. In this paper, we discuss the main indications and virtue for using CE in CD and its implementation in clinical practice.

## 2. Suspected CD

The diagnosis of IBD is based on a combination of clinical, biochemical, endoscopic and radiological characteristics. The gold standard for establishing the diagnosis in patients with suspected CD is ileocolonoscopy with biopsies. However, as 30% of lesions are exclusively located in the small bowel, the diagnosis of this sub-group may be beyond the reach of ileocolonoscopy [20,21]. These patients are typically diagnosed by cross-sectional imaging modalities. CE can visualize minor mucosal lesions that are seldom visible to radiological imaging modalities, becoming the preferred modality for patients with high clinical index of suspicion for CD and negative ileocolonoscopy [22]. Possible findings on CE suggesting a diagnosis of CD include aphthous ulcerations, linear/deep ulcerations, loss of villi, villous edema and stricture [23]. These are not specific for CD and may be seen in other types of small bowel disease, such as NSAIDS-induced enteropathy [24].

The high sensitivity of CE to detect mucosal abnormalities in the small bowel has been broadly reported [25,26,27]. In patients with suspected small bowel CD, undetected by conventional diagnostic methods, CE diagnosed CD-associated lesions at considerable rates. Ge et al. reported that CE diagnosed CD in 65% of patients suspected of having small bowel lesions [28]. In patients with suspected CD yet with normal endoscopies, CE detected lesions supporting the diagnosis of CD in 43% of patients [29]. When compared with non-invasive imaging modalities, CE has a high diagnostic yield for the detection of mucosal lesions, consistent with CD. A meta-analysis of 19 trials demonstrated a significantly increased diagnostic yield compared with radiography, CTE and even ileocolonoscopy [30]. CE can establish the diagnosis of CD in patients with small-bowel disease and normal ileocolonoscopy [31]. As for MRE, although accuracy rates are generally comparable to that of CE, several studies have found the latter to be significantly more sensitive for the detection of small bowel involvement. Jensen and colleagues reported, in a prospective blinded study of 93 patients, sensitivity and specificity rates for the diagnosis of CD of the terminal ileum as 100% and 91% by CE, compared with 81% and 86% by MRE, respectively [32]. Proximal CD was detected in eighteen patients using CE, compared with two and six patients by using MRE or CTE, respectively (*p* < 0.05). In a retrospective study by González-Suárez et al., CE had significantly higher sensitivity rates in detecting proximal and distal disease in the small bowel compared to MRE (76.6% vs. 44.7% *p* = 0.001). In the terminal ileum, CE detected lesions in 68.1% of patients, whereas MRE detected lesions in 38.3% (*p* = 0.001) [22]. These findings led to a modification of the Montreal classification in 53.1% of patients based on CE and in 12.7% of patients based on MRE (*p* < 0.05). A systematic review by Kopylov et al. revealed that CE was superior to MRE (OR 2.79; 95% CI 1.2–6.48) for the detection of proximal small bowel CD [33]. In light of these findings, CE has been implemented into consensus guidelines as a first-line diagnosis method for suspected small bowel CD and negative ileocolonoscopy [10,15].

## 3. Established CD (ECD)

Once CD is diagnosed following ileocolonoscopy, further investigation is recommended by the guidelines to assess disease extent and location (Table 1). A more proximal small bowel disease may have prognostic implications. CD particularly involving the jejunum is considered a risk factor for strictures leading to surgical interventions [34]. CTE or MRE are generally considered the first-line modalities for the evaluation of the small bowel in patients with ECD. The advantages of these modalities over CE are the ability to identify strictures, assess transmural involvement of the small bowel and avoidance of ionizing radiation exposure with MRE [23,35]. Nevertheless, as previously mentioned, CE may detect minor lesions that conventional imaging may fail to detect. When compared with both CTE and MRE, CE is significantly better in detecting proximal small bowel lesions, a finding that carries an unfavorable prognosis [36,37]. This may be pertinent in a state of CD exacerbation, despite negative results from imaging modalities. CE findings have the potential to modify treatment of ECD. Greener and colleagues demonstrated that 27% of patients with an unrecognized disease location per CE were reclassified as an advanced phenotype (B2 or B3), and at a higher rate with proximal disease, thus impacting disease management. The combination of CE and MRE changed the original Montreal classification in 49/76 patients (64%) [38]. Impact on disease management can range from change in medication dose, the initiation of biologic treatment, the avoidance of treatment in patients with complaints similar to CD but with normal CE or the avoidance of surgery [39]. Lorenzo-Zúñiga et al. evaluated the impact of CE on the management of patients with ECD. In 64% of patients, the management of CD was altered after CE, mainly by changing or adding drug therapy [40]. These treatment modifications included a change in immunomodulators and the initiation of biological agents. Calabrese and colleagues reported that CE identified small bowel non-obstructing strictures and ulcerations, providing the true extent and severity of the disease, whereas CTE significantly underestimated the extent of mucosal involvement; thus, leading to a swap in biologic therapy, avoiding the option of surgery [41]. These findings strengthen the essential role of CE to monitor disease activity in cases of established CD, as it can assess response to treatment and identify high-risk patients for disease exacerbation.

A handful of CE scoring systems for a detailed description of disease extent and severity in CD have been introduced over the years. The Capsule Endoscopy Crohn’s Disease Activity Index (CECDAI) score evaluates the degree of inflammation, disease extent and the presence of strictures, both for the proximal and distal segments of the small bowel [42,43]. The Lewis score is the most commonly used CE scoring system as it is embedded within the capsule’s software (Table 2). The small bowel is divided into three sections according to time from the first duodenal to the first cecal image, and a cumulative score is given for the presence of various findings, such as ulcers, strictures or fistulas in each segment [44,45]. A normal examination is determined for a score below/equal to 135, while a score above 790 reflects moderate to severe inflammation. This score can also aid in the diagnosis of patients with suspected CD, with a sensitivity of 82.6% and a specificity of 87.9% [46]. No differences were seen in the quantitative assessment of mucosal inflammation in ECD between these two scoring systems [47,48]. Another novel score was recently introduced for the entire digestive system for estimating mucosal inflammation using the pan-enteric PillCam Crohn’s capsule (Table 3) [49].

## 4. ECD—Treat to Target

To date, an important treatment goal in patients with ECD is mucosal healing [50]. In small bowel disease, where ileocolonoscopy is limited, response to treatment may be evaluated by various imaging modalities, including CTE, MRE, intestinal US or CE [17]. The recently published STRIDE-II guidelines confirmed the STRIDE-I long-term targets of clinical remission and endoscopic healing as the treat-to-target goals of ECD treatment [51,52]. These underscore the shifting pendulum from not only clinical, but rather to a combination of biomarker and endoscopic remission.

The potential role of CE for treat-to-target monitoring in patients with small bowel CD has been evaluated in several studies. In 2014, a prospective study of patients with ECD showed a 50% rate of complete mucosal healing after 52 weeks of treatment with Adalimumab. In the colon, the time to mucosal healing was shorter compared to mucosal healing of the small bowel [18,53]. Kopylov and colleagues reported that 33% of patients with small bowel CD in clinical and biomarker remission had mucosal healing on CE examination [11]. A meta-analysis demonstrated that patients with mucosal healing detected by CE had a significant OR (11.1) for improved outcomes after 12–24 months [54]. Nakamura and colleagues demonstrated an 80% improvement of the Lewis score in patients with active small bowel disease (LS > 135) who received additional medications and underwent a follow-up CE [55]. Improvement in the Lewis score was also seen in 72% of the asymptomatic group who received additional medications [55]. In 2018, Melmed and colleagues showed the high correlation between ileocolonoscopy scores and CE scores for the assessment of mucosal disease activity over a 6-month period [56]. Overall, these findings show that CE is probably the best tool to reliably assess mucosal healing as part of the treat-to-target strategy in the small bowel CD.

## 5. ECD—Predicting Relapse before and after Surgery

The role of CE in ECD is further expanded as CE can monitor disease flares and predict the future (short- or long-term) course of the disease. As such, a prospective observational cohort study in 2019 applied an intensive monitoring strategy during a 24-month follow-up period in patients with quiescent CD [57]. In this study, a baseline small bowel CE Lewis score of 350 or more identified patients with a future flare (area under the curve 0.79, *p* < 0.0001) and an increase of 383 points from baseline predicted short-term (within 6 months) risk exacerbation (area under the curve 0.79, *p* = 0.011). A score below 350 was associated with prolonged remission (negative predictive value of 92%). These findings suggest that CE can be an accurate monitoring tool for small bowel CD by predicting future short- and long-term disease course.

CE can also be used to monitor post-operative CD patients and should be taken into account for post-operative recurrence when ileocolonoscopy is unsuccessful [58,59]. One-year post-operative recurrence of CD after ileal resection can occur in up to 70% of cases [60]. The sensitivity of CE in diagnosing post-operative recurrence has been demonstrated in several studies. A small study from 2014 showed that post-operative lesions occurred in 78% of patients 2–3 weeks after surgery for CD [61]. Yung and colleagues demonstrated, in a meta-analysis, the diagnostic role of CE in the assessment of post-operative endoscopic recurrence in CD. The pooled sensitivity for CE in post-operative endoscopic recurrence in this study was 100% [62]. Post-operative lesions detected by CE can impact clinical management and outcomes in asymptomatic patients with CD. This was observed in a group of 37 patients, of whom CE detected endoscopic recurrence in 11 patients missed by ileocolonoscopy. In addition, one-year remission rates were maintained in all patients with CE-identified remission, thus eliminating the need for pharmacologic prophylaxis [63].

Shiga and colleagues recently reported that patients who underwent post-operative follow-up CE had significantly lower risk of hospitalization, repeat surgery or need for endoscopic dilation compared with patients who did not have CE follow-up [64]. In summary, CE is a useful non-invasive method that may increase the diagnostic accuracy in post-operative CD patients suspected of disease recurrence, with a potential to modify treatment [65].

## 6. IBD Unclassified

CE can assist in cases of IBD unclassified/indeterminate colitis. It is possible that patients with ulcerative colitis (UC) can be reclassified as having CD and vice versa [66]. With considerable reclassification rates, small bowel lesions detected by CE can diagnose CD in patients with IBD unclassified/indeterminate colitis.

Considering the presence of at least three small bowel ulcers as a diagnostic criterion, 16.7% to 38.9% of IBD unclassified patients were eventually diagnosed with CD using CE [67,68]. In pediatric IBD unclassified patients, small bowel lesions typical of CD were detected in 43.8% of those undergoing CE [69]. These rates are even higher for symptomatic IBD patients with pouchitis. In a retrospective cohort study, 65.2% of patients who were diagnosed with pouchitis following ileo-anal anastomosis had CE consisting of typical CD findings [70].

## 7. Retention Risk of CE

Capsule retention in the gastrointestinal tract is the main complication of CE, rarely leading to small-bowel obstruction. The risk of retention is increased in patients with a stricturing phenotype, or in patients with a history of small bowel obstruction or past abdominal surgery. Retention rates reported from two meta-analyses ranged from 2.3% to 3.6% in suspected CD, and from 4.6% to 8.2% in patients with ECD [71]. Real world studies have shown retention rates around 2% in ECD as well.

Capsule retention is usually an asymptomatic episode. According to the European Society of Gastrointestinal Endoscopy guidelines, in the event of an asymptomatic capsule retention, the recommended management is observation [72]. Up to 50% of capsule retention patients spontaneously excrete the capsule without further management after 15 days, usually 4–12 weeks after ingestion. In addition, a trial of steroids may allow capsule excretion. However, if this fails to succeed, capsules are removed by device-assisted enteroscopy or surgical removal after 3–6 months, or if patients experience symptoms of acute obstruction [73,74].

To ensure a safe passage of the small bowel, an ingestible radio-opaque patency capsule (PC) of the same dimensions as the CE is administered prior to the CE. The PC self-dissolves in the bowel in the case of retention, therefore being potentially less harmful than the CE [75]. As per the updated European Society of Gastrointestinal Endoscopy guidelines, the use of a PC before small-bowel capsule endoscopy is recommended in patients with ECD to decrease the capsule retention rate [76]. A meta-analysis by Zhang et al. demonstrated a sensitivity of 97% and a specificity of 83% for PC in diagnosing small bowel retention [77]. A retrospective study from 2011 compared the performance of the PC and imaging in the detection of clinically significant small bowel strictures, showing similar sensitivity (57% vs. 71%; *p* = 1.00) and specificity (86% vs. 97%; *p* = 0.22) [78]. In a recent meta-analysis, patients with ECD had capsule retention rates of 2.32% after negative small bowel cross-sectional imaging and 2.88% after negative PC [79]. Although these rates are somewhat similar, a PC is essential to rule out strictures in patients at increased risk of capsule retention, even in the presence of a negative small bowel cross-sectional imaging. This was demonstrated by Rondonotti et al., who showed comparable capsule retention rates in low-risk patients (20/2942; 0.7%) and high-risk patients (0.7%, 1/151) with negative PC [80]. However, high-risk patients with negative cross-sectional imaging (CTE or MRE) had significantly higher capsule retention rates (8.3%, 2/24; *p* = 0.049).

In addition, in quiescent CD, PC has a potential prognostic role in identifying patients who are high-risk for future complications. In a post-hoc analysis of two prospective cohort studies of quiescent small bowel CD, patients with a failed PC (PC retention) had higher rates of a need for intestinal surgery or endoscopic dilation during a 34-median-month follow-up (21.3% vs. 1.4%, 4.4–93.7, *p* < 0.001) compared to patients with a passed PC [81]. These patients were also prone to more admissions (23.3% vs. 5.7%, *p* < 0.001), and clinical flares (43.9% vs. 27.7%, *p* = 0.005) during follow-up [73].

A limitation of the patency capsule can be the cost; however, using a selective approach can be cost-effective when compared with other strategies (avoiding the use of patency capsules or using patency for all referrals prior to CE). An additional consideration is an incomplete small-bowel examination leading to failure of CE to visualize the whole small bowel. This can occur due to a stricture in the small bowel or a limited battery life. Battery life can be a limiting factor during capsule endoscopies, and 16.5% of studies are incomplete due to battery expiration. In the past, CE was only able to visualize up to 80% of the small bowel extent due to battery consumption. However, battery life in the new generation of capsules can last up to 11–14 h, practically allowing full visualization of the small bowel in all patients.

## 8. Pan-Enteric Capsule Endoscopy (PCC)

**Figure 1:**
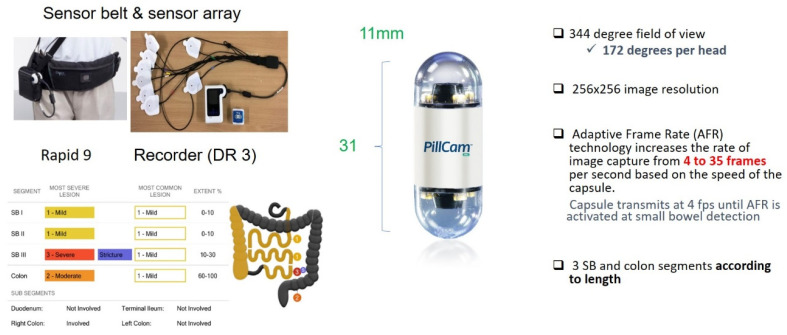
The PillCam Crohn’s capsule.

Crohn’s disease is a pan-enteric disease that requires a tool that can investigate the entire bowel. The advancement of video capsule endoscopy through the years has led to a high diagnostic yield of lesions in the small intestine. The introduction of pan-enteric capsule endoscopy has enabled the visualization of the large bowel and spares the need for diagnostic colonoscopy. In 2009, the second-generation colon capsule endoscopy was developed (PillCam Colon 2; Medtronic), displaying an improved accuracy method for visualizing the colon and detecting colonic lesions [82]. The utilization of the PillCam Colon 2 as an endoscopy tool for the whole digestive tract in CD later lead to the introduction of a novel capsule from Medtronic—the PillCam Crohn’s capsule. This is a true mouth-to-anus pan-enteric video capsule system that enables visualization of the small and large bowels, allowing evaluation of the disease extent and severity [83]. This capsule was designed with a longer (~14 h) battery life, two wide-angle (336-degree) cameras and an adapted frame rate, similar to the PillCam Colon 2 (Figure 1). A CD-dedicated software (Rapid 9) enables a real-time acquisition of disease activity in three segments of the small bowel and the colon, defining the most severe lesion, the most common lesion and the extent of the disease in each segment. As data are saved in the Rapid software, a comparison of different examinations after different treatments is possible. For an accurate quantitative score of inflammation using this novel pan enteric capsule, a recently proposed score was introduced to monitor pan-enteric mucosal inflammation in CD (Table 3). This score correlated well with the Lewis score (area under the curve 0.9; *p* < 0.0001), with excellent reliability [49].

Leighton and colleagues, using the PCC without its specific software, demonstrated an improved performance of the PCC compared with ileocolonoscopy among 66 CD patients with active disease who underwent both procedures [84]. Both per-subject diagnostic yield and the per-segment diagnostic yield rate for active CD lesions were higher in the PCC compared with ileocolonoscopy. A substantial rate of active lesions (18%) was only detected by the PCC, thus concluding that PCC should be at least a complementary procedure to ileocolonoscopy among patients with CD. Bruining et al. prospectively assessed 99 patients with non-stricturing disease in terms of the performance of PCC and its software compared with ileocolonoscopy and/or MRE, in the detection of mucosal lesions [85]. The authors demonstrated comparable sensitivity rates and higher specificity rates in the overall intestinal assessment between the PCC compared to the MRE and/or ileocolonoscopy. PCC had higher sensitivity and specificity in the proximal small bowel compared with MRE, as well as in the terminal ileum compared with MRE and/or ileocolonoscopy, and had equal performance in the colon compared to ileocolonoscopy. In addition to these impressive disease-detecting capabilities, patient satisfaction was superior for CE compared to the two other procedures. These findings emphasized the great advantage of PCC to enable a reliable disease staging with a single procedure.

## 9. Monitoring

Monitoring disease activity is fundamental in ECD. Volkers et al. explored the endoscopic response to infliximab in 22 patients using PCC. They gave the capsule before, 8 and 12 weeks after treatment; as a result, 27% of patients observed endoscopic remission and another 50% observed endoscopic response [86]. Similarly, a prospective study of patients treated with Vedolizumab undergoing a PCC exam (before treatment initiation, week 14 and 52 of therapy) is being currently conducted [87]. Interim results from this study show a 35–50% improvement in inflammatory scores (Lewis score; Eliakim score), as well as calprotectin at week 14.

## 10. Treat to Target

A study by Tai et al. examined PCC performance in real life and assessed whether it caused treatment modifications among 93 patients with CD [88]. PCC detected active disease in (67.6%) with ECD. Disease extent was upstaged in (33%) patients, nine with newly upper GIT involvement. PCC findings led to treatment intensification in (39%) patients, and it was associated with proximal small-bowel involvement. Symptoms or biochemical/fecal markers were inferior in identifying active CD compared with PCC. Oliva et al. assessed the yield of PCC among 48 pediatric patients [89]. PCC detected significant inflammation in 71% of patients involving the small bowel or the colon, leading to treatment change in 71% of them at baseline and in another 23% at 24 weeks follow-up, thus increasing the mucosal healing rate to 58% at week 52.

Based on Ben-Horin’s findings on the predictive potential of CE, a prospective randomized controlled trial was proposed and has recently finished recruitment: the CURE-CD: Comprehensive individUalized pRoactive ThErapy of Crohn’s Disease [90]. Based on their inflammatory score, patients with ECD in clinical remission are divided into three groups: a low-risk group (LS < 350), designed for follow-up and a high-risk group (LS >350). The high-risk group is randomly divided into standard versus proactive (optimal) care. The primary endpoint being the rate of clinical relapse or disease complications at 24 months. Patients are monitored by PCC every 6 months, yearly by MRE and by biomarkers every 3 months.

## 11. Post-Operative Crohn’s Disease

Hausmann et al. conducted a prospective multi-center pilot study assessing the value of PCC. They performed the study using PillCam Colon 2 as their pan-enteric capsule, post-operatively for 4–8 weeks and 4–8 months [91]. Twenty-two patients were included. Significant disease was found in 19% of patients at the early stage and in 50% of patients at the late stage (Rutgeerts > 2). Ileocolonoscopy revealed significant inflammation in 33% of patients.

## 12. Artificial Intelligence in CE

Recently, much progress has been made in artificial intelligence (AI) deep learning algorithms as they have led to transformations in various medical fields, including gastroenterology. AI implemented in CE image detection analysis has the potential to reduce reading times and limit inter-observer variability. An increasing number of studies have been published regarding the potential of AI in the field of CE. A systematic review by Mohan et al. initially assessed 4245 studies. The review eventually included nine studies in the final analysis of patients with IBD and non-IBD patients [92]. Overall, the pooled accuracy of CE in detecting ulcers or bleeding in patients with IBD was 95.4% (sensitivity 95.5%, specificity 95.8%, positive predictive value 95.8% and negative predictive value 96.8%). In a meta-analysis from 2020 that included 19 retrospective studies of deep learning applications in CE, detection accuracy rates were above 90% for the majority of studies. Pooled sensitivity and specificity for ulcer detection were 0.95 (95% confidence interval [CI], 0.89–0.98) and 0.94 (95% CI, 0.90–0.96), respectively [93]. A study by Klang and colleagues demonstrated high accuracy rates for the automated detection of mucosal ulcers on 17,640 CE images of 49 CD patients. The area under the curve (AUC) for the detection of CD ulcers by randomly split images was 0.99, and accuracies ranged from 95.4% to 96.7%. For individual patient-level experiments, the AUCs were 0.94–0.99 [94]. These high accuracy rates were demonstrated in the detection of CD-associated strictures on CE images as well. The AUC for the differentiation between strictures and normal mucosa was 0.98, and between strictures and all ulcers was 0.942 [95].

In summary, the use of AI has already been applied in various medical analysis fields and it has the potential to show great promise in the clinical practice of gastroenterology and IBD [19]. The implementation of integrated algorithms in CE can improve accuracy and shorten reading times. It appears that, in the near future, it will play a fundamental role and become a new standard for automated lesion detection and characterization in CD.

## 13. Conclusions

In conclusion, CE is a minimally invasive and accurate modality which plays an important role for the detection and management of CD in the small bowel and colon. Based on current guidelines, the main indications of CE in CD include the evaluation of disease classification, severity and extent in suspected or confirmed ECD, the monitoring of mucosal healing and the prediction of relapse. The introduction of pan-enteric capsule enables visualizing the whole digestive tract. The PillCam Crohn’s, a novel pan-enteric capsule for the diagnosis and management of CD, is a true mouth-to-anus pan-enteric video capsule system. It is useful in demonstrating disease extent and severity, and monitoring pan-enteric disease activity as part of treat-to-target strategy. In addition, it allows for the prediction of clinical and post-operative relapse, and may allow medical intervention so that these are prevented.

## Figures and Tables

**Table 1:** When do we usually employ endoscopy in Crohn’s disease?

Initial diagnosis Monitoring activity, severity and extent Assess response to treatment→Mucosal healing Predicting relapse Post operative recurrence Survey for CRC in longstanding disease Treating IBD complications (stricture etc.)

**Table 2:** The Lewis inflammatory score—embedded within PillCam software.

Villous appearance	Normal-0	Short segment-8	Single-1
	Edematous-1	Long segment-12	Patchy-14
		Whole tertile-20	Diffuse-17
Ulcer	None-0	Short segment-5	<1/4-9
	Single-3	Long segment-10	1/4–1/2-12
	Few-5	Whole tertile-15	>1/2-18
	Multiple-10		
Stenosis (whole study)			
Stenosis	None	Ulcerated-24	Traversed-7
	Single-14	Nonulcerated-2	Not traversed-10
	Multiple-20		
Gralnek et al. [44]			
Small bowel divided into 3 tertiles according to small bowel passage time			
N < 135; 135–790 = mild; >790 = moderate-severe inflammation			

**Table 3:** PillCam CD Inflammatory score—Eliakim score.

**A. Most common lesion (MCL)**	
none	0
mild	1
moderate	2
severe	3
**B. Most severe lesion (MSL)**	
none	0
mild	1
moderate	2
severe	3
**C. Extent of disease**	
none	0
0–10%	1
10–30%	2
30–60%	3
60–100%	4
**D. Stricture**	
none	0
one traversed	1
>1 traversed	2
Retention	3
Segmental score: [(A + B) × C] + D	
Small bowel PCC (PCCS-SB): SB1 + SB2 + SB3	
Panenteric PCC (PCCS): SB1 + SB2 + SB3 + RC + LC	

## Data Availability

No new data were created or analyzed in this study.

## References

[1] Iddan G., Meron G., Glukhovsky A., Swain P. (2000). Wireless capsule endoscopy. Nature.

[2] Davies G.R., Benson M.J., Gertner D.J., Van Someren R.M.N., Rampton D.S., Swain C.P. (1995). Diagnostic and therapeutic push type enteroscopy in clinical use. Gut.

[3] Yamamoto H., Yano T., Kita H., Sunada K., Ido K., Sugano K., Costamagna G., Riccioni M.E. (2003). New system of double-balloon enteroscopy for diagnosis and treatment of small intestinal disorders. Gastroenterology.

[4] Akerman P.A., Agrawal D., Cantero D., Pangtay J. (2008). Spiral enteroscopy with the new DSB overtube: A novel technique for deep peroral small-bowel intubation. Endoscopy.

[5] Neuhaus H., Beyna T., Schneider M., Devière J. (2016). Novel motorized spiral enteroscopy: First clinical case. Gastrointest. Endosc..

[6] Murphy K.P., McLaughlin P.D., O’Connor O.J., Maher M.M. (2014). Imaging the small bowel. Curr. Opin. Gastroenterol..

[7] Hong S.M., Jung S.H., Baek D.H. (2021). Diagnostic yields and clinical impacts of capsule endoscopy. Diagnostics.

[8] Kopylov U., Seidman E.G. (2015). Diagnostic modalities for the evaluation of small bowel disorders. Curr. Opin. Gastroenterol..

[9] Eliakim R. (2008). Video capsule endoscopy of the small bowel. Curr. Opin. Gastroenterol..

[10] Pennazio M., Spada C., Eliakim R., Keuchel M., May A., Mulder C.J., Rondonotti E., Adler S.N., Albert J., Baltes P. (2015). Small-bowel capsule endoscopy and device-assisted enteroscopy for diagnosis and treatment of small-bowel disorders: European Society of Gastrointestinal Endoscopy (ESGE) Clinical Guideline. Endoscopy.

[11] Kopylov U., Yablecovitch D., Lahat A., Neuman S., Levhar N., Greener T., Klang E., Rozendorn N., Amitai M.M., Ben-Horin S. (2015). Detection of Small Bowel Mucosal Healing and Deep Remission in Patients with Known Small Bowel Crohn’s Disease Using Biomarkers, Capsule Endoscopy, and Imaging. Am. J. Gastroenterol..

[12] Cosnes J., Gowerrousseau C., Seksik P., Cortot A. (2011). Epidemiology and natural history of inflammatory bowel diseases. Gastroenterology.

[13] Torres J., Mehandru S., Colombel J.F., Peyrin-Biroulet L. (2017). Crohn’s disease. Lancet.

[14] Louis E., Collard A., Oger A.F., Degroote E., Aboul Nasr El Yafi F., Belaiche J. (2001). Behaviour of Crohn’s disease according to the Vienna classification: Changing pattern over the course of the disease. Gut.

[15] Maaser C., Sturm A., Vavricka S.R., Kucharzik T., Fiorino G., Annese V., Calabrese E., Baumgart D.C., Bettenworth D., Borralho Nunes P. (2019). ECCO-ESGAR Guideline for Diagnostic Assessment in IBD Part 1: Initial diagnosis, monitoring of known IBD, detection of complications. J. Crohn’s Colitis.

[16] Lamb C.A., Kennedy N.A., Raine T., Hendy P.A., Smith P.J., Limdi J.K., Hayee B., Lomer M.C.E., Parkes G.C., Selinger C. (2019). British Society of Gastroenterology consensus guidelines on the management of inflammatory bowel disease in adults. Gut.

[17] Efthymiou A., Viazis N., Mantzaris G., Papadimitriou N., Tzourmakliotis D., Raptis S., Karamanolis D.G. (2008). Does clinical response correlate with mucosal healing in patients with Crohn’s disease of the small bowel? A prospective, case-series study using wireless capsule endoscopy. Inflamm. Bowel Dis..

[18] Hall B., Holleran G., Chin J.L., Smith S., Ryan B., Mahmud N., McNamara D. (2014). A prospective 52 week mucosal healing assessment of small bowel Crohn’s disease as detected by capsule endoscopy. J. Crohn’s Colitis.

[19] Tontini G.E., Rimondi A., Vernero M., Neumann H., Vecchi M., Bezzio C., Cavallaro F. (2021). Artificial intelligence in gastrointestinal endoscopy for inflammatory bowel disease: A systematic review and new horizons. Therap. Adv. Gastroenterol..

[20] Cleynen I., González J.R., Figueroa C., Franke A., McGovern D., Bortlík M., Crusius B.J.A., Vecchi M., Artieda M., Szczypiorska M. (2013). Genetic factors conferring an increased susceptibility to develop Crohn’s disease also influence disease phenotype: Results from the IBDchip European Project. Gut.

[21] Dulai P.S., Singh S., Vande Casteele N., Boland B.S., Rivera-Nieves J., Ernst P.B., Eckmann L., Barrett K.E., Chang J.T., Sandborn W.J. (2019). Should We Divide Crohn’s Disease Into Ileum-Dominant and Isolated Colonic Diseases?. Clin. Gastroenterol. Hepatol..

[22] González-Suárez B., Rodriguez S., Ricart E., Ordás I., Rimola J., Díaz-González Á., Romero C., De Miguel C.R., Jáuregui A., Araujo I.K. (2018). Comparison of Capsule Endoscopy and Magnetic Resonance Enterography for the Assessment of Small Bowel Lesions in Crohn’s Disease. Inflamm. Bowel Dis..

[23] Bourreille A., Ignjatovic A., Aabakken L., Loftus E.V., Eliakim R., Pennazio M., Bouhnik Y., Seidman E., Keuchel M., Albert J.G. (2009). Role of small-bowel endoscopy in the management of patients with inflammatory bowel disease: An international OMED-ECCO consensus. Endoscopy.

[24] Graham D.Y., Opekun A.R., Willingham F.F., Qureshi W.A. (2005). Visible small-intestinal mucosal injury in chronic NSAID users. Clin. Gastroenterol. Hepatol..

[25] Koulaouzidis A., Rondonotti E., Karargyris A. (2013). Small-bowel capsule endoscopy: A ten-point contemporary review. World J. Gastroenterol..

[26] Tukey M., Pleskow D., Legnani P., Cheifetz A.S., Moss A.C. (2009). The utility of capsule endoscopy in patients with suspected Crohn’s disease. Am. J. Gastroenterol..

[27] Doherty G.A., Moss A.C., Cheifetz A.S. (2011). Capsule endoscopy for small-bowel evaluation in Crohn’s disease. Gastrointest. Endosc..

[28] Ge Z.Z., Hu Y.B., Xiao S.D. (2004). Capsule endoscopy in diagnosis of small bowel Crohn’s disease. World J. Gastroenterol..

[29] Herrerías J.M., Caunedo A., Rodríguez-Téllez M., Pellicer F., Herrerías J.M. (2003). Capsule endoscopy in patients with suspected Crohn’s disease and negative endoscopy. Endoscopy.

[30] Dionisio P.M., Gurudu S.R., Leighton J.A., Leontiadis G.I., Fleischer D.E., Hara A.K., Heigh R.I., Shiff A.D., Sharma V.K. (2010). Capsule endoscopy has a significantly higher diagnostic yield in patients with suspected and established small-bowel crohn’s disease: A meta-analysis. Am. J. Gastroenterol..

[31] Leighton J.A., Gralnek I.M., Cohen S.A., Toth E., Cave D.R., Wolf D.C., Mullin G.E., Ketover S.R., Legnani P.E., Seidman E.G. (2014). Capsule endoscopy is superior to small-bowel follow-through and equivalent to ileocolonoscopy in suspected Crohn’s disease. Clin. Gastroenterol. Hepatol..

[32] Jensen M.D., Nathan T., Rafaelsen S.R., Kjeldsen J. (2011). Diagnostic Accuracy of Capsule Endoscopy for Small Bowel Crohn’s Disease Is Superior to That of MR Enterography or CT Enterography. Clin. Gastroenterol. Hepatol..

[33] Kopylov U., Yung D.E., Engel T., Vijayan S., Har-Noy O., Katz L., Oliva S., Avni T., Battat R., Eliakim R. (2017). Diagnostic yield of capsule endoscopy versus magnetic resonance enterography and small bowel contrast ultrasound in the evaluation of small bowel Crohn’s disease: Systematic review and meta-analysis. Dig. Liver Dis..

[34] Lazarev M., Huang C., Bitton A., Cho J.H., Duerr R.H., McGovern D.P., Proctor D.D., Regueiro M., Rioux J.D., Schumm P.P. (2013). Relationship between proximal Crohn’s disease location and disease behavior and surgery: A cross-sectional study of the IBD Genetics Consortium. Am. J. Gastroenterol..

[35] Moy M.P., Sauk J., Gee M.S. (2016). The role of MR enterography in assessing Crohn’s disease activity and treatment response. Gastroenterol. Res. Pract..

[36] Voderholzer W.A., Beinhoelzl J., Rogalla P., Murrer S., Schachschal G., Lochs H., Ortner M.A. (2005). Small bowel involvement in Crohn’s disease: A prospective comparison of wireless capsule endoscopy and computed tomography enteroclysis. Gut.

[37] Petruzziello C., Onali S., Calabrese E., Zorzi F., Ascolani M., Condino G., Lolli E., Naccarato P., Pallone F., Biancone L. (2010). Wireless capsule endoscopy and proximal small bowel lesions in Crohn’s disease. World J. Gastroenterol..

[38] Greener T., Klang E., Yablecovitch D., Lahat A., Neuman S., Levhar N., Avidan B., Yanai H., Dotan I., Chowers Y. (2016). The impact of magnetic resonance enterography and capsule endoscopy on the re-classification of disease in patients with known Crohn’s Disease: A prospective Israeli IBD research nucleus (IIRN) study. J. Crohn’s Colitis.

[39] De Bona M., Bellumat A., Cian E., Valiante F., Moschini A., De Boni M. (2006). Capsule endoscopy findings in patients with suspected Crohn’s disease and biochemical markers of inflammation. Dig. Liver Dis..

[40] Lorenzo-Zúñiga V., De Vega V.M., Domènech E., Cabré E., Mañosa M., Boix J. (2010). Impact of capsule endoscopy findings in the management of Crohn’s Disease. Dig. Dis. Sci..

[41] Calabrese C., Dussias N., Impellizzeri G., Lauro A., Pagano N. (2021). CEing More—Assessing Small Bowel Crohn’s with Capsule Endoscopy (CE). Dig. Dis. Sci..

[42] Gal E., Geller A., Fraser G., Levi Z., Niv Y. (2008). Assessment and validation of the new capsule endoscopy Crohn’s disease activity index (CECDAI). Dig. Dis. Sci..

[43] Niv Y., Ilani S., Levi Z., Hershkowitz M., Niv E., Fireman Z., Odonnel S., Omorain C., Eliakim R., Scapa E. (2012). Validation of the Capsule Endoscopy Crohns Disease Activity Index (CECDAI or Niv score): A multicenter prospective study. Endoscopy.

[44] Gralnek I.M., Defranchis R., Seidman E., Leighton J.A., Legnani P., Lewis B.S. (2008). Development of a capsule endoscopy scoring index for small bowel mucosal inflammatory change. Aliment. Pharmacol. Ther..

[45] Cotter J., Dias De Castro F., Magalhães J., João Moreira M., Rosa B. (2015). Validation of the Lewis score for the evaluation of small-bowel Crohn’s disease activity. Endoscopy.

[46] Rosa B., Moreira M.J., Rebelo A., Cotter J. (2011). Lewis Score: A useful clinical tool for patients with suspected Crohn’s Disease submitted to capsule endoscopy. J. Crohn's Colitis.

[47] Yablecovitch D., Lahat A., Neuman S., Levhar N., Avidan B., Ben-Horin S., Eliakim R., Kopylov U. (2018). The Lewis score or the capsule endoscopy Crohn’s disease activity index: Which one is better for the assessment of small bowel inflammation in established Crohn’s disease?. Therap. Adv. Gastroenterol..

[48] Koulaouzidis A., Douglas S., Plevris J.N. (2012). Lewis score correlates more closely with fecal calprotectin than capsule endoscopy crohn’s disease activity index. Dig. Dis. Sci..

[49] Eliakim R., Yablecovitch D., Lahat A., Ungar B., Shachar E., Carter D., Selinger L., Neuman S., Ben-Horin S., Kopylov U. (2020). A novel PillCam Crohn’s capsule score (Eliakim score) for quantification of mucosal inflammation in Crohn’s disease. United Eur. Gastroenterol. J..

[50] Reinink A.R., Lee T.C., Higgins P.D.R. (2016). Endoscopic Mucosal Healing Predicts Favorable Clinical Outcomes in Inflammatory Bowel Disease: A Meta-analysis. Inflamm. Bowel Dis..

[51] Peyrin-Biroulet L., Sandborn W., Sands B.E., Reinisch W., Bemelman W., Bryant R.V., D’Haens G., Dotan I., Dubinsky M., Feagan B. (2015). Selecting Therapeutic Targets in Inflammatory Bowel Disease (STRIDE): Determining Therapeutic Goals for Treat-to-Target. Am. J. Gastroenterol..

[52] Turner D., Ricciuto A., Lewis A., D’Amico F., Dhaliwal J., Griffiths A.M., Bettenworth D., Sandborn W.J., Sands B.E., Reinisch W. (2021). STRIDE-II: An Update on the Selecting Therapeutic Targets in Inflammatory Bowel Disease (STRIDE) Initiative of the International Organization for the Study of IBD (IOIBD): Determining Therapeutic Goals for Treat-to-Target strategies in IBD. Gastroenterology.

[53] Hall B.J., Holleran G.E., Smith S.M., Mahmud N., McNamara D.A. (2014). A prospective 12-week mucosal healing assessment of small bowel Crohn’s disease as detected by capsule endoscopy. Eur. J. Gastroenterol. Hepatol..

[54] Niv Y. (2017). Small-bowel mucosal healing assessment by capsule endoscopy as a predictor of long-term clinical remission in patients with Crohn’s disease: A systematic review and meta-analysis. Eur. J. Gastroenterol. Hepatol..

[55] Nakamura M., Yamamura T., Maeda K., Sawada T., Mizutani Y., Ishikawa T., Furukawa K., Ohno E., Kawashima H., Miyahara R. (2018). Validity of Capsule Endoscopy in Monitoring Therapeutic Interventions in Patients with Crohn’s Disease. J. Clin. Med..

[56] Melmed G.Y., Dubinsky M.C., Rubin D.T., Fleisher M., Pasha S.F., Sakuraba A., Tiongco F., Shafran I., Fernandez-Urien I., Rosa B. (2018). Utility of video capsule endoscopy for longitudinal monitoring of Crohn’s disease activity in the small bowel: A prospective study. Gastrointest. Endosc..

[57] Ben-Horin S., Lahat A., Amitai M.M., Klang E., Yablecovitch D., Neuman S., Levhar N., Selinger L., Rozendorn N., Turner D. (2019). Assessment of small bowel mucosal healing by video capsule endoscopy for the prediction of short-term and long-term risk of Crohn’s disease flare: A prospective cohort study. lancet. Gastroenterol. Hepatol..

[58] Buisson A., Chevaux J.B., Bommelaer G., Peyrin-Biroulet L. (2012). Diagnosis, prevention and treatment of postoperative Crohn’s disease recurrence. Dig. Liver Dis..

[59] Bourreille A., Jarry M., D’Halluin P.N., Ben-Soussan E., Maunoury V., Bulois P., Sacher-Huvelin S., Vahedy K., Lerebours E., Heresbach D. (2006). Wireless capsule endoscopy versus ileocolonoscopy for the diagnosis of postoperative recurrence of Crohn’s disease: A prospective study. Gut.

[60] Rutgeerts P., Geboes K., Vantrappen G., Beyls J., Kerremans R., Hiele M. (1990). Predictability of the postoperative course of Crohn’s disease. Gastroenterology.

[61] Kono T. (2014). Prospective postsurgical capsule endoscopy in patients with Crohn’s disease. World J. Gastrointest. Endosc..

[62] Yung D.E., Har-Noy O., Tham Y.S., Ben-Horin S., Eliakim R., Koulaouzidis A., Kopylov U. (2018). Capsule Endoscopy, Magnetic Resonance Enterography, and Small Bowel Ultrasound for Evaluation of Postoperative Recurrence in Crohn’s Disease: Systematic Review and Meta-Analysis. Inflamm. Bowel Dis..

[63] Han Z.M., Qiao W.-g., Ai X.-y., Li A.-m., Chen Z.-y., Feng X.-c., Zhang J., Wan T.-m., Xu Z.-m., Bai Y. (2018). Impact of capsule endoscopy on prevention of postoperative recurrence of Crohn’s disease. Gastrointest. Endosc..

[64] Shiga H., Abe I., Kusaka J., Shimoyama Y., Moroi R., Kuroha M., Kakuta Y., Kinouchi Y., Masamune A. (2022). Capsule Endoscopy Is Useful for Postoperative Tight Control Management in Patients with Crohn’s Disease. Dig. Dis. Sci..

[65] Mir A., Nguyen V.Q., Soliman Y., Sorrentino D. (2021). Wireless capsule endoscopy for diagnosis and management of post-operative recurrence of crohn’s disease. Life.

[66] Moum B., Ekbom A., Vatn M.H., Aadland E., Sauar J., Lygren I., Schulz T., Stray N., Fausa O. (1997). Inflammatory bowel disease: Re-evaluation of the diagnosis in a prospective population based study in south eastern Norway. Gut.

[67] Maunoury V., Savoye G., Bourreille A., Bouhnik Y., Jarry M., Sacher-Huvelin S., Soussan E.B., Lerebours E., Galmiche J.P., Colombel J.F. (2007). Value of wireless capsule endoscopy in patients with indeterminate colitis (inflammatory bowel disease type unclassified). Inflamm. Bowel Dis..

[68] Lopes S., Figueiredo P., Portela F., Freire P., Almeida N., Lérias C., Gouveia H., Leitão M.C. (2010). Capsule endoscopy in inflammatory bowel disease type unclassified and indeterminate colitis serologically negative. Inflamm. Bowel Dis..

[69] Di Nardo G., Oliva S., Ferrari F., Riccioni M.E., Staiano A., Lombardi G., Costamagna G., Cucchiara S., Stronati L. (2011). Usefulness of wireless capsule endoscopy in paediatric inflammatory bowel disease. Dig. Liver Dis..

[70] Long M.D., Barnes E., Isaacs K., Morgan D., Herfarth H.H. (2011). Impact of capsule endoscopy on management of inflammatory bowel disease: A single tertiary care center experience. Inflamm. Bowel Dis..

[71] Rezapour M., Amadi C., Gerson L.B. (2017). Retention associated with video capsule endoscopy: Systematic review and meta-analysis. Gastrointest. Endosc..

[72] Rondonotti E., Spada C., Adler S., May A., Despott E.J., Koulaouzidis A., Panter S., Domagk D., Fernandez-Urien I., Rahmi G. (2018). Small-bowel capsule endoscopy and device-assisted enteroscopy for diagnosis and treatment of small-bowel disorders: European Society of Gastrointestinal Endoscopy (ESGE) Technical Review. Endoscopy.

[73] Nemeth A., Wurm Johansson G., Nielsen J., Thorlacius H., Toth E. (2017). Capsule retention related to small bowel capsule endoscopy: A large European single-center 10-year clinical experience. United Eur. Gastroenterol. J..

[74] Rondonotti E. (2017). Capsule retention: Prevention, diagnosis and management. Ann. Transl. Med..

[75] Nemeth A., Kopylov U., Koulaouzidis A., Wurm Johansson G., Thorlacius H., Amre D., Eliakim R., Seidman E.G., Toth E. (2016). Use of patency capsule in patients with established Crohn’s disease. Endoscopy.

[76] Pennazio M., Rondonotti E., Despott E.J., Dray X., Keuchel M., Moreels T., Sanders D.S., Spada C., Carretero C., Cortegoso Valdivia P. (2023). Small-bowel capsule endoscopy and device-assisted enteroscopy for diagnosis and treatment of small-bowel disorders: European Society of Gastrointestinal Endoscopy (ESGE) Guideline—Update 2022. Endoscopy.

[77] Zhang W., Han Z.L., Cheng Y., Xu Y.Z., Xiao K., Li A.M., Wang Y.D., Li Y., Liu S. (2014). De Value of the patency capsule in pre-evaluation for capsule endoscopy in cases of intestinal obstruction. J. Dig. Dis..

[78] Yadav A., Heigh R.I., Hara A.K., Decker G.A., Crowell M.D., Gurudu S.R., Pasha S.F., Fleischer D.E., Harris L.A., Post J. (2011). Performance of the patency capsule compared with nonenteroclysis radiologic examinations in patients with known or suspected intestinal strictures. Gastrointest. Endosc..

[79] Pasha S.F., Pennazio M., Rondonotti E., Wolf D., Buras M.R., Albert J.G., Cohen S.A., Cotter J., D’Haens G., Eliakim R. (2020). Capsule Retention in Crohn’s Disease: A Meta-analysis. Inflamm. Bowel Dis..

[80] Rondonotti E., Soncini M., Girelli C.M., Russo A., De Franchis R. (2016). Negative small-bowel cross-sectional imaging does not exclude capsule retention in high-risk patients. Eur. J. Gastroenterol. Hepatol..

[81] Ukashi O., Kopylov U., Ungar B., Haj-Natour O., Selinger L., Neuman S., Yanai H., Dotan I., Yablecovitch D., Lahat A. (2023). Patency Capsule: A Novel Independent Predictor for Long-Term Outcomes Among Patients With Quiescent Crohn’s Disease. Am. J. Gastroenterol..

[82] Eliakim R., Yassin K., Niv Y., Metzger Y., Lachter J., Gal E., Sapoznikov B., Konikoff F., Leichtmann G., Fireman Z. (2009). Prospective multicenter performance evaluation of the second-generation colon capsule compared with colonoscopy. Endoscopy.

[83] Eliakim R., Spada C., Lapidus A., Eyal I., Pecere S., Fernández-Urién I., Lahat A., Costamagna G., Schwartz A., Ron Y. (2018). Evaluation of a new pan-enteric video capsule endoscopy system in patients with suspected or established inflammatory bowel disease—Feasibility study. Endosc. Int. Open.

[84] Leighton J.A., Helper D.J., Gralnek I.M., Dotan I., Fernandez-Urien I., Lahat A., Malik P., Mullin G.E., Rosa B. (2017). Comparing diagnostic yield of a novel pan-enteric video capsule endoscope with ileocolonoscopy in patients with active Crohn’s disease: A feasibility study. Gastrointest. Endosc..

[85] Bruining D.H., Oliva S., Fleisher M.R., Fischer M., Fletcher J.G. (2020). Panenteric capsule endoscopy versus ileocolonoscopy plus magnetic resonance enterography in Crohn’s disease: A multicentre, prospective study. BMJ Open Gastroenterol..

[86] Volkers A., Bossuyt P., de Jong J., Pouillon L., Gecse K., Duijvestein M., Ponsioen C., D’Haens G., Löwenberg M. (2022). Assessment of endoscopic response using pan-enteric capsule endoscopy in Crohn’s disease; the Sensitivity to Change (STOC) study. Scand. J. Gastroenterol..

[87] Kopylov U., Lipkin M., Ungar B., Haj O., Shachar E., Lahat A., Ben-Horin S., Eliakim R. (2022). P160 Pan-enteric mucosal inflammation in CD patients treated with vedolizumab—Interim results of a prospective observational study using a panenteric capsule (PiilCam CD). J. Crohn’s Colitis.

[88] Tai F.W.D., Ellul P., Elosua A., Fernandez-Urien I., Tontini G.E., Elli L., Eliakim R., Kopylov U., Koo S., Parker C. (2021). Panenteric capsule endoscopy identifies proximal small bowel disease guiding upstaging and treatment intensification in Crohn’s disease: A European multicentre observational cohort study. United Eur. Gastroenterol. J..

[89] Oliva S., Aloi M., Viola F., Mallardo S., Civitelli F., Maccioni F., Hassan C., Papoff P., Cucchiara S., Cohen S.A. (2019). A Treat to Target Strategy Using Panenteric Capsule Endoscopy in Pediatric Patients With Crohn’s Disease. Clin. Gastroenterol. Hepatol..

[90] Shomron B.-H. Comprehensive Individualized pRoactive ThErapy of Crohn’s Disease Trial: The CURE-CD Trial. Clin Identifier NCT03555058. NCT03555058.

[91] Hausmann J., Schmelz R., Walldorf J., Filmann N., Zeuzem S., Albert J.G. (2017). Pan-intestinal capsule endoscopy in patients with postoperative Crohn’s disease: A pilot study. Scand. J. Gastroenterol..

[92] Mohan B.P., Khan S.R., Kassab L.L., Ponnada S., Chandan S., Ali T., Dulai P.S., Adler D.G., Kochhar G.S. (2021). High pooled performance of convolutional neural networks in computer-aided diagnosis of GI ulcers and/or hemorrhage on wireless capsule endoscopy images: A systematic review and meta-analysis. Gastrointest. Endosc..

[93] Soffer S., Klang E., Shimon O., Nachmias N., Eliakim R., Ben-Horin S., Kopylov U., Barash Y. (2020). Deep learning for wireless capsule endoscopy: A systematic review and meta-analysis. Gastrointest. Endosc..

[94] Klang E., Barash Y., Margalit R.Y., Soffer S., Shimon O., Albshesh A., Ben-Horin S., Amitai M.M., Eliakim R., Kopylov U. (2020). Deep learning algorithms for automated detection of Crohn’s disease ulcers by video capsule endoscopy. Gastrointest. Endosc..

[95] Klang E., Grinman A., Soffer S., Margalit Yehuda R., Barzilay O., Amitai M.M., Konen E., Ben-Horin S., Eliakim R., Barash Y. (2021). Automated Detection of Crohn’s Disease Intestinal Strictures on Capsule Endoscopy Images Using Deep Neural Networks. J. Crohn’s Colitis.

